# Efficient Training on Alzheimer’s Disease Diagnosis with Learnable Weighted Pooling for 3D PET Brain Image Classification

**DOI:** 10.3390/electronics12020467

**Published:** 2023-01-16

**Authors:** Xin Xing, Muhammad Usman Rafique, Gongbo Liang, Hunter Blanton, Yu Zhang, Chris Wang, Nathan Jacobs, Ai-Ling Lin

**Affiliations:** 1 Department of Computer Science, University of Kentucky,Lexington, KY 40506, USA; 2 Department of Radiology, University of Missouri, Columbia, MO 65212, USA; 3 Kitware Inc., Clifton Park, NY 12065, USA; 4 Department of Computing and Cyber Security, Texas A & M University-San Antonio, San Antonio, TX 78224, USA; 5 Department of Computer Science, University of Missouri, Columbia, MO 65211, USA; 6 Department of Computer Science & Engineering, Washington University in St. Louis, St. Louis, MO 63130, USA; 7 Department of Biological Sciences, University of Missouri, Columbia, MO 65211, USA; 8 Institute for Data Science and Informatics, University of Missouri, Columbia, MO 65211, USA

**Keywords:** efficient training, Alzheimer’s disease, deep learning, 3D-to-2D module

## Abstract

Three-dimensional convolutional neural networks (3D CNNs) have been widely applied to analyze Alzheimer’s disease (AD) brain images for a better understanding of the disease progress or predicting the conversion from cognitively impaired (CU) or mild cognitive impairment status. It is well-known that training 3D-CNN is computationally expensive and with the potential of overfitting due to the small sample size available in the medical imaging field. Here we proposed a novel 3D-2D approach by converting a 3D brain image to a 2D fused image using a Learnable Weighted Pooling (LWP) method to improve efficient training and maintain comparable model performance. By the 3D-to-2D conversion, the proposed model can easily forward the fused 2D image through a pre-trained 2D model while achieving better performance over different 3D and 2D baselines. In the implementation, we chose to use ResNet34 for feature extraction as it outperformed other 2D CNN backbones. We further showed that the weights of the slices are location-dependent and the model performance relies on the 3D-to-2D fusion view, with the best outcomes from the coronal view. With the new approach, we were able to reduce 75% of the training time and increase the accuracy to 0.88, compared with conventional 3D CNNs, for classifying amyloid-beta PET imaging from the AD patients from the CU participants using the publicly available Alzheimer’s Disease Neuroimaging Initiative dataset. The novel 3D-2D model may have profound implications for timely AD diagnosis in clinical settings in the future.

## 1. Introduction

Alzheimer’s disease (AD) is the most common form of dementia and the sixth leading cause of death in the U.S. [1]. The major pathological hallmarks of AD are amyloid-beta (*Aβ*) plaques (A), hyperphosphorylated neurofibrillary tau (T) tangles, and neurodegeneration (N), known as the A/T/N framework, a descriptive classification scheme for AD biomarkers [2–4]. Non-invasive neuroimaging has been used for the early detection of A/T/N changes. In particular, positron emission tomography (PET) has been used to image *Aβ* for detecting AD pathology progression [5–8].

Recent works on AD classification are mainly based on 3D CNN models. Based on Ref. [9], the current 3D model methods are divided into three categories: (1) the 3D regions of interest (ROI) based CNN models, (2) the 3D patch-level CNN models, and (3) the 3D subject-level CNN models. The 3D regions of interest (ROI) based CNN models take ROIs of the 3D brain image as the input to train the 3D CNN model [10,11]. This approach is time-consuming because it requires manually drawn ROIs. The 3D patch-level CNN models may include Refs. [12,13], which extract the 27 patches of 3D image and independently train 27 3D CNNs for ensemble prediction. It is still a complex model since 27 individual 3D CNN models are trained together. The 3D subject-level CNN models the whole 3D brain images as input of the 3D CNN architectures. For instance, some works [14,15] have proposed to forward the whole 3D brain image as input of the 3D CNN architectures, which shows high accuracy classification without the need for manual feature extraction. While these 3D CNN models achieve excellent performance, these methods have shown two limitations. First, compared to 2D-CNN, training the 3D-CNN model is computationally expensive. Second, directly training a deep learning model with a relatively small size of the medical image dataset could lead to overfitting. When working with 2D medical images, this can be addressed by using transfer learning, such as adopting widely used ImageNet pre-trained CNN models [16]. Unfortunately, such models are not readily available for 3D datasets. These two limitations impede the applications for AD diagnosis and predicting AD progression using 3D imaging data.

Other than the 3D CNN method, researchers also explore using 3D images with 2D CNNs. The initial method is to pick the slice of 3D volumes as the input of the model. Ozsahin et al. [17] proposed selecting and converting a 2D PET slice into a vector as the input of a multilayer perceptron. Ghaffari et al. [18] applied the transfer learning approach for MRI image classification. Odusami et al. [19,20] proposed a hybrid CNN model by parallelly combining the ResNet18 and DenseNet121 together as feature extractors and concatenating the extracted features for prediction. Another common approach is to use temporal pooling to convert a video clip into a 2D image. The idea is to distill a video into a 2D motion representation that summarizes the whole video clip. Dynamic Image [21,22] is one of the most famous methods along this line of research. Ref. [21] proposed to use an approximate rank pooling (ARP) operation to convert the video frames into a 2D dynamic image. In 3D medical image applications, Liang et al. [23] combined 2D breast mammography and 3D breast tomosynthesis by using ARP on the 3D volumes. Xing et al. [24] applied ARP on the 3D brain MRI images for Alzheimer’s disease classification. In general, ARP achieves good performance on various tasks. However, as a fixed function, the weight of each frame in APR is deterministic and is calculated using only the frame index and the total number of frames.

To overcome the limitations, we propose to convert 3D volumes into 2D images with a Learnable Weighted Pooling (LWP) method. The advantage of LWP is that it computes the weighted values of each slide of the 3D image, and provides an overall weighted slice as a fusion image. By converting to a 2D image, we were able to dramatically shorten the training time and apply multiple pre-trained 2D models that are currently not available for 3D CNN, such as VGGNet [25], ResNet [26], DenseNet [27], MobileNet [28], and EfficientNet [29]. The benefits of using these pre-trained models will allow us to optimize the choice of the feature extractor for different datasets.

In this study, we applied and compared the results between these widely used and pre-trained 2D-CNN models. We further incorporated an attention module for the classifier, strengthening discriminative feature learning and enhancing the deep learning model performance. We hypothesized that by converting 3D PET-*Aβ* imagery to 2D, we were able to reduce the training time while enhancing the performance for AD prediction. We consider our main contributions as follows:

- We proposed a novel learnable weighted pooling module for 3D-to-2D image projection and end-to-end network architecture;
- We employed a new dual-attention mechanism module on the top of 2D CNNs to boost the model performance;
- Compared with 3D CNNs models, the proposed model gained comparable performance with less training computation cost;
- We conducted an intensive evaluation with multiple imaging modalities among different 3D-to-2D modules.

## 2. Materials and Methods

### 2.1. Data

We obtained the PET-*Aβ* (AV45) imaging from the Alzheimer’s Disease Neuroimaging Initiative (ADNI) database [30]. Participants were required to have baseline *Aβ* imaging biomarkers (from Florbetapir AV45 PET). Each subject in ADNI may have multiple neuroimaging scans at different time points. We used the first-time scan of each subject for the early diagnosis task. The collected PET images are pre-processed from the “Coreg, Avg, Std Img and VoxSiz, Uniform Resolution” category. The PET image size is 96 × 160 × 160. Table 1 shows the demographics of the CU and AD participants. The dataset in total includes 381 subjects, with 214 CU subjects and 167 AD subjects. The two study groups were balanced in gender, race, and age (CU: 73.6 ± 6.0, AD: 74.7 ± 8.4, *p*-value = 0.1511), but not education. CU overall had longer education (in years) than AD participants. Notably, the groups differed in terms of the expression of the ε4 allele of apolipoprotein E (APOE *ε*4), the largest genetic risk factor for Alzheimer’s disease, with the AD group being significantly more likely to carry APOE *ε*4 than CU subjects [31–33].

### 2.2. Architecture

**Figure 1.**
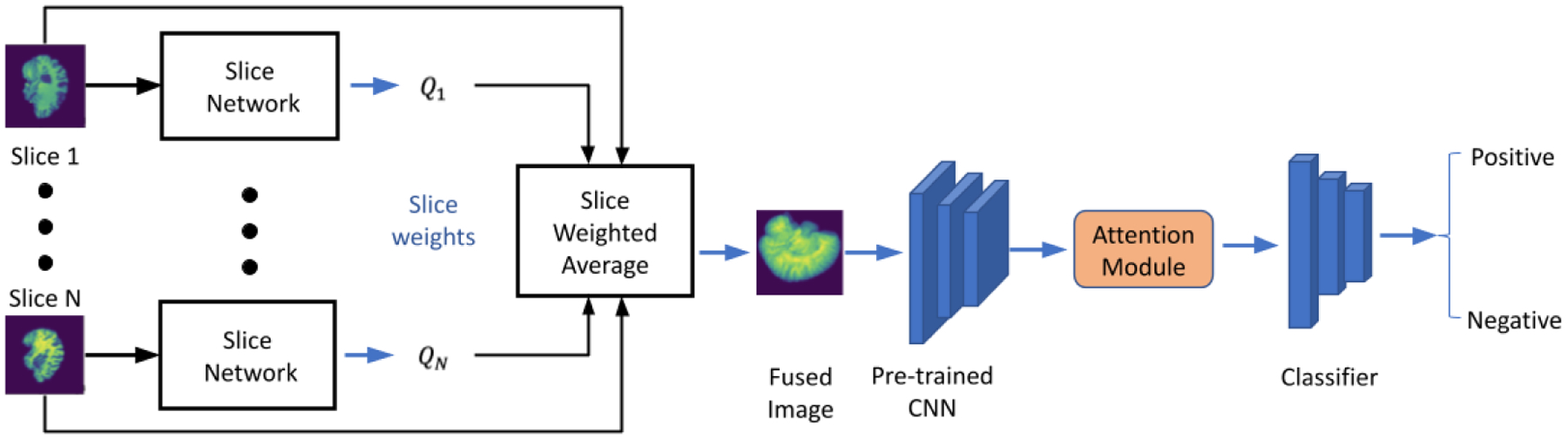
The workflow of our proposed CNN model.

Figure 1 shows the overall workflow of the proposed model. First, each slice of the 3D medical image was passed to the slice network that converts 3D images to a 2D fused image by fusing all the slices using a pooling method. Then, we forwarded the 2D fused image through a pre-trained feature extractor. Afterward, we passed the extracted feature to a dual-attention mechanism to boost our model performance. Finally, the output of the attention module was forwarded to a shallow classifier built by fully connected layers for diagnosis prediction.

### 2.3. Learnable Weighted Pooling (LWP)

Given a 3D image $V=\left[I_{1},I_{2},\ldots ,I_{T}\right]\in {\mathbb{R}}^{T\times H\times W}$, where *I*_*x*_ is a 2D slice $\in {\mathbb{R}}^{H\times W}$ and *T* is equal to the number of slices. A 3D-to-2D projection aims to fuse all slices of the 3D image to get a 2D image $\in {\mathbb{R}}^{H\times W}$. Inspired by the ARP [21], we proposed the LWP method. For the LWP operation on a 3D image *V* with T slices *I*_1_, …, *I*_*T*_, the CNN built slice network *ψ* outputs for the corresponding weight of each slice *I*_*x*_ as *Q*_*x*_ = *ψ*(*I*_*x*_). We use the softmax function *σ*, to normalize the slice weight *α*_*t*_ between 0 and 1 as Equation (1). The fused 2D image $F_{I_{1}},\ldots ,I_{t}$ is the sum of all weighted slices over a certain view dimension:

(1) $$\sigma \left(Q_{x}\right)=\frac{e^{Q_{x}}}{\sum_{x=1}^{T}e^{Qx}}$$

(2) $$F_{I_{1},\ldots ,I_{t}}=\sum_{x=1}^{T}I_{x}\cdot \sigma \left(Q_{x}\right)$$

**Figure 2.**
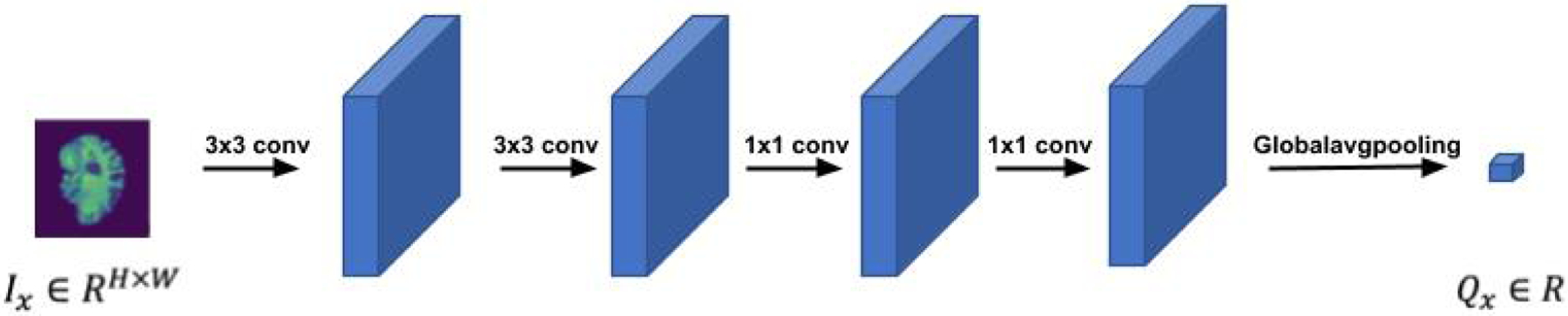
The structure of the slice network.

Figure 2 depicts the LWP structure. The value *Q*_*x*_ of a single input slice *I*_*x*_ ∈ *R*^*H×W*^ is a scalar, *Q*_*x*_ *∈ R*, which means that the temporal rank pooling is on an image level operation. We forward a single slice through a shallow CNN model, which includes four convolutional layers and a global average pooling layer to get a single scalar per slice. The parameters of the slice network were initialized by kaiming initialization and are fine-tuned during the training.

The whole idea is a 3D-to-2D image-level projection by sum fusion of the weighted slices. There are several significant differences between LWP and ARP. First, the ARP slice weight *α*_*t*_ of each slice is only related to the total slice number *T* and current slice index *α*_*t*_ = 2*t − T −* 1, which is fixed and not learnable. Second, ARP applies an average of slices up to slice *t* on each slice $\theta \left(I_{t}\right)=\frac{1}{t}\sum_{x=1}^{T}I_{\tau }$. To address the limitations of ARP, we propose a flexible and trainable fusion module, LWP.

### 2.4. Attention Module and Classifier

**Figure 3.**
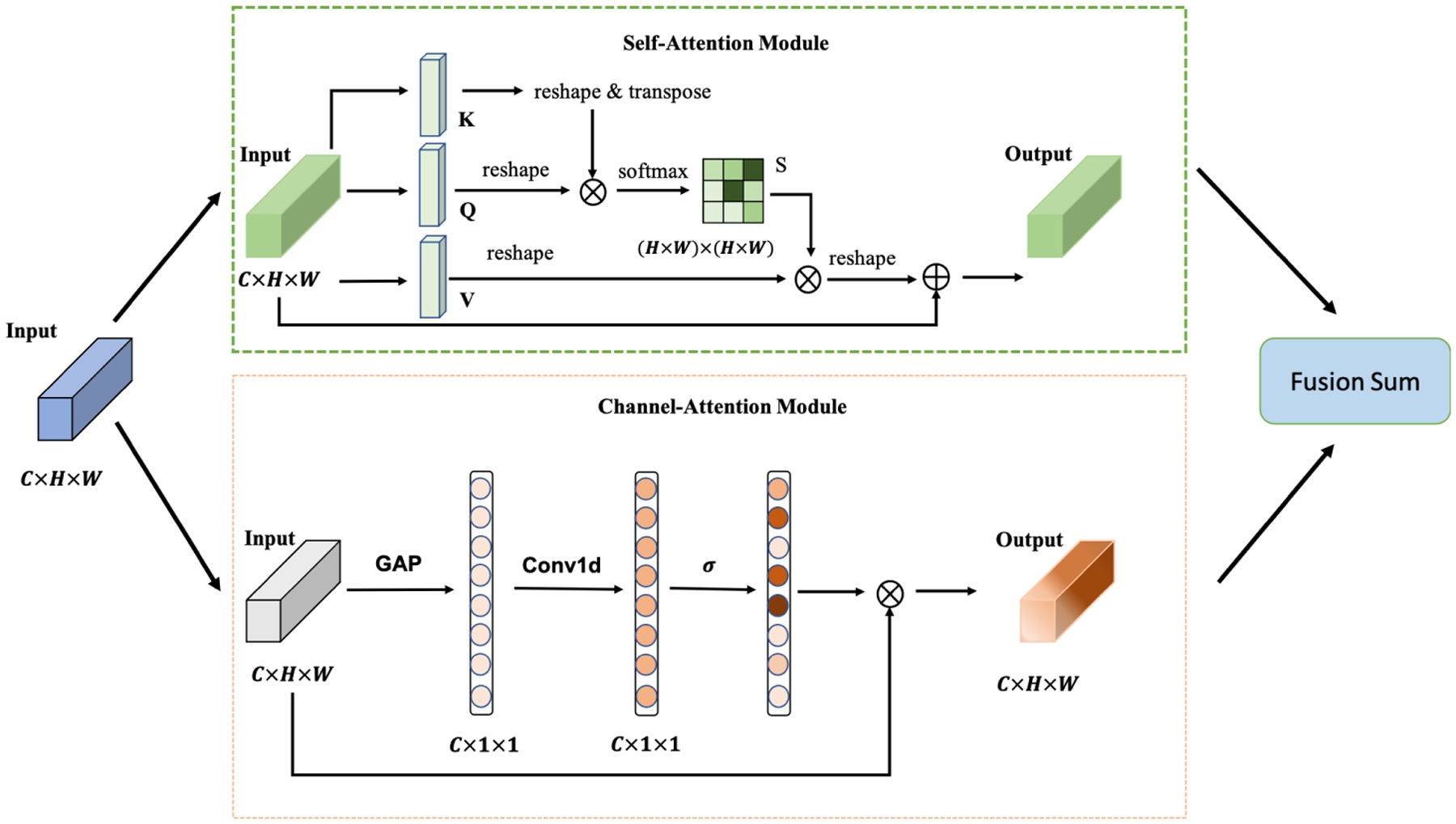
The structure of the attention module. The input of the attention module is the extracted features of the CNN backbone. The output of the attention module is forwarded to the classifier.

We deploy the attention module to simultaneously refine our extracted feature spatial-wise and channel-wise, so we adopt a dual-attention mechanism architecture. To simplify our model architecture, we employ the dual-attention module on the top of the CNN feature extractor. Figure 3 depicts the structure of the attention mechanism. The dual-attention module structure is similar to Ref. [34]. It contains two sub-modules: the self-attention (SA) module [35] as the position-wise correlation computation and the channel-attention (CA) module [36] to calculate the channel-wise correlation. The CA module of Ref. [34] needs quadratic computation cost, but the channel-wise attention of our module is calculated by a Conv1d operation, which is more cost-efficient. We forward the input feature *Input* ∈ ℛ^𝒞×ℋ×𝒲^ through the two sub-modules respectively and use sum fusion to merge as the final output feature *Output* ∈ ℛ^𝒞×ℋ×𝒲^.

Our classifier contains three fully connected (FC) layers. The output dimensions of the three FC layers are 512, 64, and 2. Batch normalization and dropout layers are attached after the first two layers. The dropout probability is 0.5.

### 2.5. Loss Function

In previous AD classification studies, work was mainly concentrated on binary classification. In our work, we did the same for ease of comparison. The overall loss function is weighted binary cross-entropy. For a 3D image *V* with label *l* and probability prediction *p*(*l|V*), the loss function is:

(3) $$\text{loss}\;(l,V)=w_{p}l\;\text{log}\;(p(l|V))+w_{n}(1-l)\;\text{log}\;(1-p(l|V))$$

where the label *l* = 0 indicates a negative sample and *l* = 1 indicates a positive sample and *w*_*p*_ and *w*_*n*_ are loss weights for the positive and negative samples, respectively.

## 3. Results

To evaluate the proposed method, we carried out several experiments on 3D PET images. The experimental results demonstrated that LWP achieves better performance than the baselines. In the following subsections, we first introduce the implementation details and evaluation metrics, then we report our results on the PET image dataset. Finally, we perform a series of ablation experiments on our dual-attention mechanism.

### 3.1. Implementation and Metrics

We implemented the CNN models using PyTorch. We trained and tested the models using the 5-fold cross-validation. The feature extractors were pre-trained on ImageNet [37]. The weights of the classifier were randomly initialized. Both the feature extractor and classifier were fine-tuned during the training. For the 2D models, we set the batch size to 16. Adam optimizer with *beta*1 = 0.9, *beta*2 = 0.999, and learning rate of 1 ×10^−4^ was used during the training. For the 3D CNN models, we followed the parameters setting of Ref. [14,15]. We trained all the models for 150 epochs. We computed loss weights for positive (*w*_*p*_) and negative (*w*_*n*_) classes based on the dataset distribution, by using *w*_*p*_ = 1.28, *w*_*n*_ = 1.

A 3D brain image may be viewed from three directions: axial, coronal, and sagittal. In the implementation, we took the LWP operation on a coronal view for the PET image. More details can be found in Section 3.2.2.

To evaluate the performance of our model, we used accuracy (Acc), area under the curve of Receiver Operating Characteristics (AUC), F1 score (F1), Precision, Recall, and Average Precision (AP) as our evaluation metrics. We evaluated the training computation cost by the average epoch training time (e-Time). The accuracy is calculated with the following Equation (4):

(4) $$\text{Accuracy }=\frac{TP+TN}{TP+TN+FP+FN}$$

where *TP* is the True Positive, *TN* is the True Negative, *FP* is the False Positive, and *FN* is the False Negative.

The *precision* is calculated by the following Equation (5):

(5) $$\text{precision }=\frac{TP}{TP+FP}$$

The *recall* is calculated by the following Equation (6):

(6) $$\text{recall}=\frac{TP}{TP+FN}$$

The *F*1 is calculated by the following Equation (7):

(7) $$F1=2\times \frac{\text{ precision }\cdot \text{ recall }}{\text{ precision }+\text{ recall }}$$

The AUC curves compare the true positive rate and the false positive rate at different decision thresholds. AP summarizes a precision-recall curve as the weighted mean of precision achieved at each threshold.

### 3.2. Evaluation

#### 3.2.1. Feature Extractor

High-quality feature extraction is crucial for the final prediction. Different pre-trained CNN models can output different features in terms of size and effective receptive field. Ke et al. [38] concluded that architecture improvements on ImageNet may not lead to improvement in medical imaging tasks. We conducted five different pre-trained CNNs on the medical dataset to determine which CNN models perform best for our task. To simplify the experiment, we set the PET-AV45 image as the input dataset using LWP and excluded the attention module from the whole experiment. Table 2 shows different CNN models and the corresponding final metrics. Considering the accuracy and AUC, the ResNet34 delivers comparable performance. In the following experiment, we used ResNet34 as our feature extractor.

#### 3.2.2. Axial vs. Coronal vs. Sagittal

**Figure 4.**
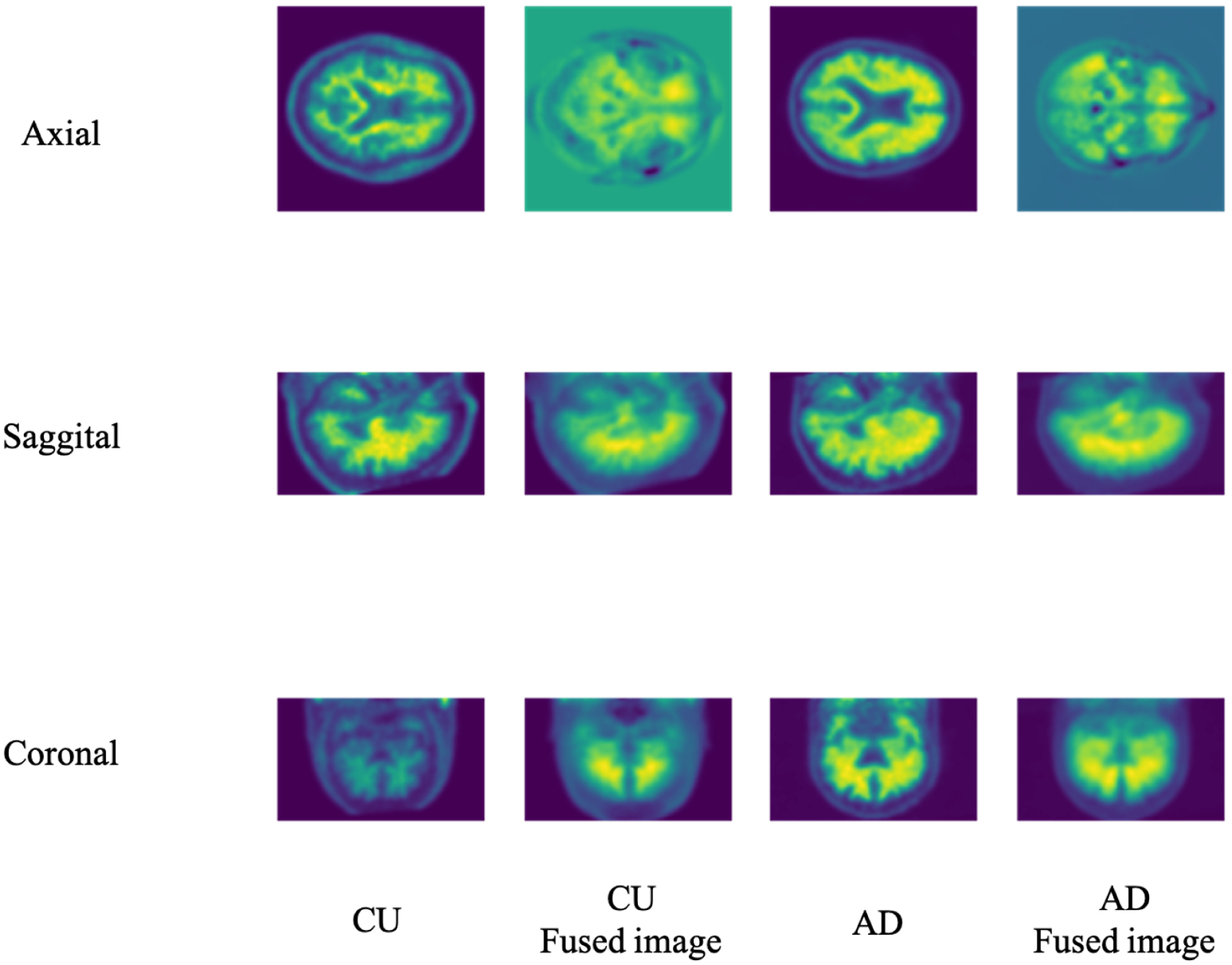
The different views of the 3D Brain image slice and the corresponding 3D-to-2D fused image. Based on the axial, sagittal, and coronal views, the 3D PET image size is 96 × 160 × 160. The corresponding fusion images of PET under different views are different: 160 × 160 of axial view, 96 × 160 of sagittal view, and 96 × 160 of coronal view.

There are three standard views of 3D imaging in medical imaging: sagittal, coronal, and axial. In the main paper, we selected the coronal view for PET empirically. The remainder of this section summarizes the experiment we conducted. Figure 4 illustrates the different views of the 3D PET images. Considering that the LWP performance may vary due to the different views fusion, we conducted experiments to specify the view influence on our model performance. Table 3 shows the model performance on the three views under the imaging modality. As a PET image, the coronal view fusion offers better performance than others. Therefore, our following experiments set the fusion views as the default of our LWP model without specific mention.

#### 3.2.3. CU vs. AD

**Figure 5.**
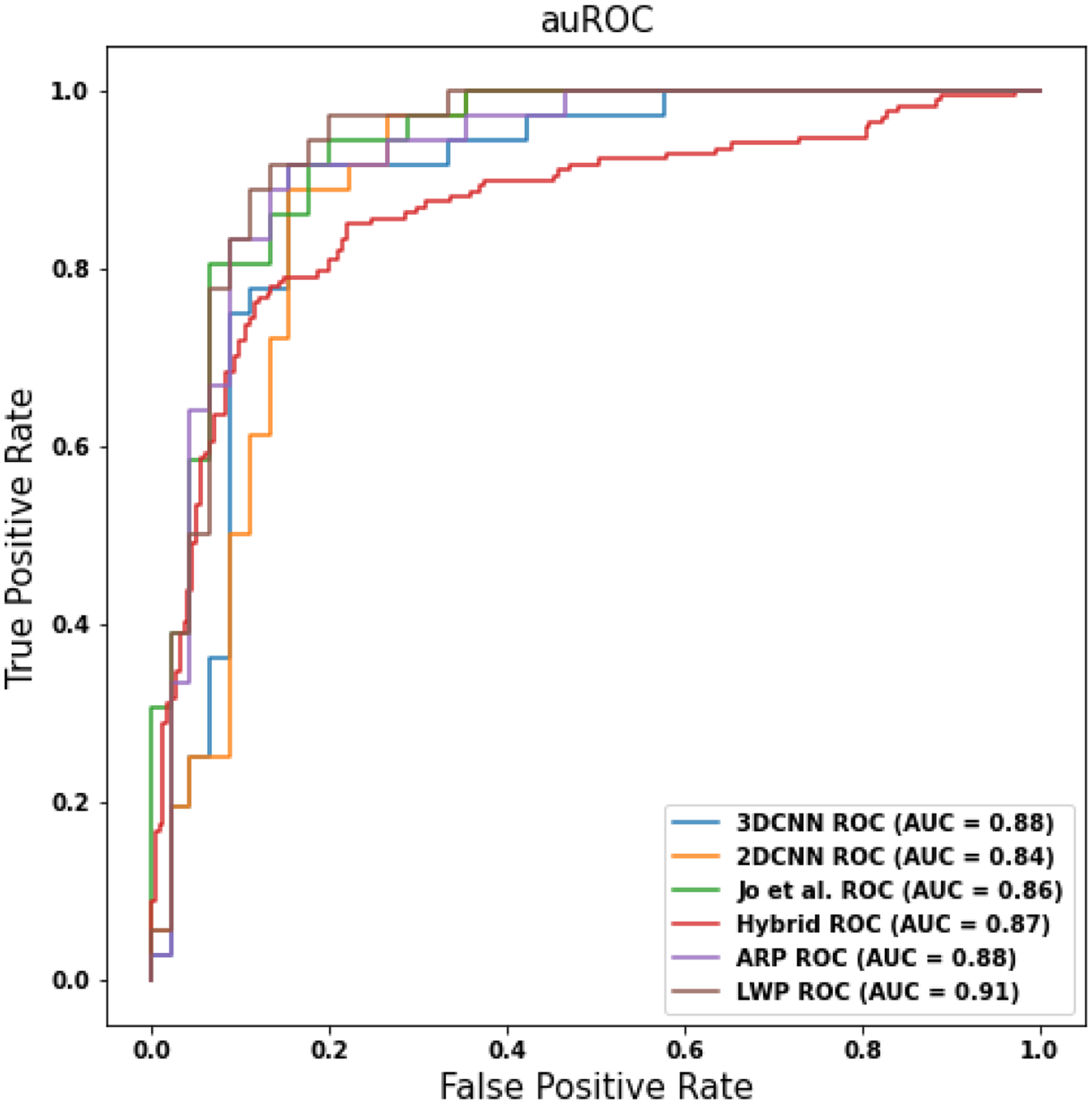
The ROC curves of the different models.

**Figure 6.**
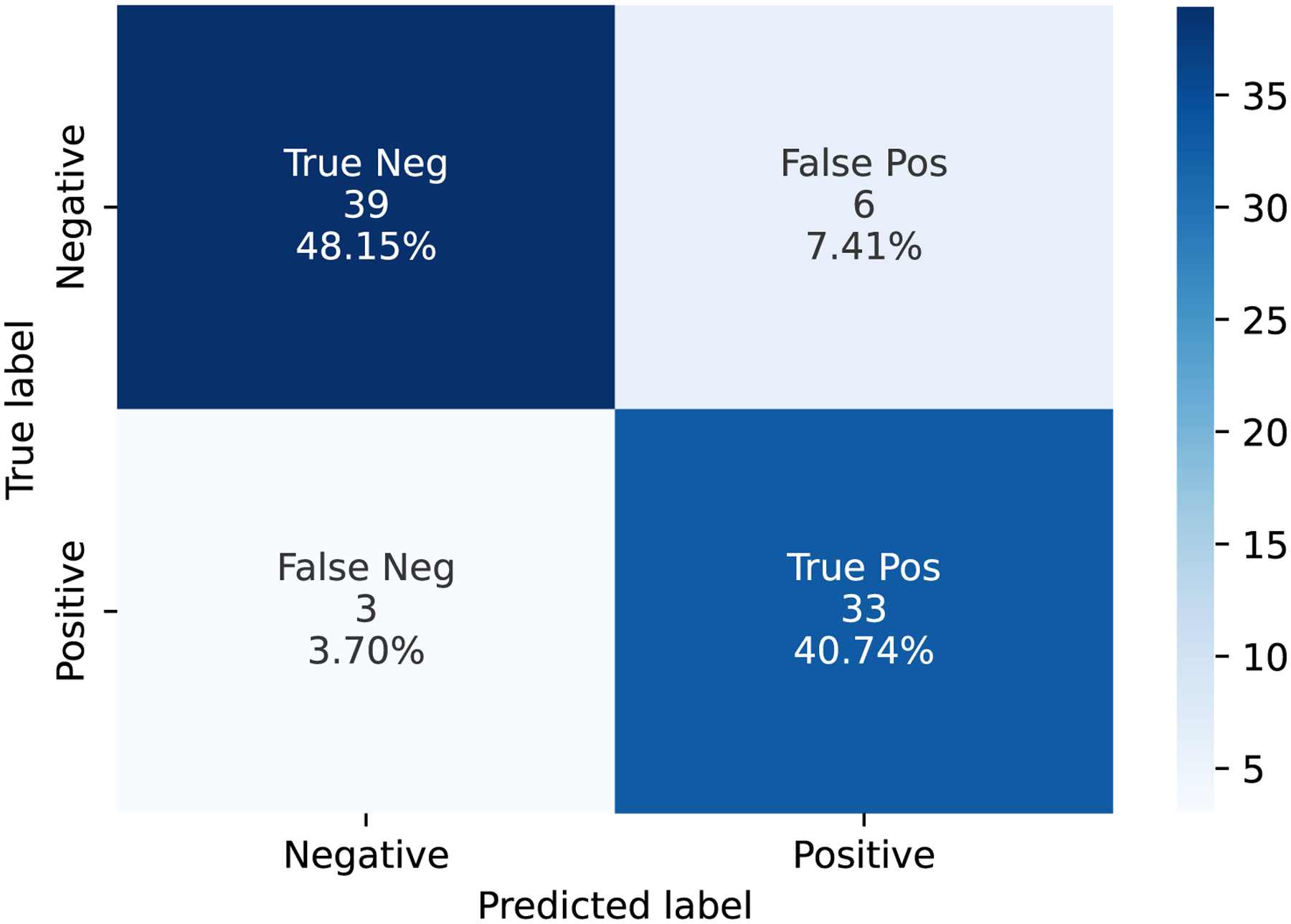
The confusion matrix of LWP on the test data.

We conducted the binary classification on the CU and AD prediction. To compare the pooling operation, we set three pooling baselines, namely Max pooling (Max.), Average pooling (Avg.), and ARP. Table 4 presents the experiment results on different models, showing that the proposed 3D-to-2D model outperforms the other CNN models. Compared with the 3D CNN baseline model, the LWP method improves 4.6% in accuracy (from 0.84 to 0.88) and 3.3% in AUC (from 0.87 to 0.90), respectively. We used the Multiply-Add operations (MADs) and training epoch time (e-time) as the reference, considering the training computation cost. Table 5 shows the training computation cost of different CNN models. The e-time of the LWP is around 26.0% of the 3D baseline model, and the MADs of LWP is around 19.0% of the 3D baseline model. We further conducted the t-test on the e-time between LWP and the 3D CNN models, the *p*-value is <0.0001, proving the significant improvement of the efficient training. Furthermore, we showed the AUC graph as Figure 5 and the confusion matrix as Figure 6. In Figure 6, “Negative” indicates CU subjects, while “Positive” indicates AD subjects.

#### 3.2.4. Attention Mechanism Ablation Study

We evaluated the ablation study to specify our dual-attention module. The baseline (BS) model structure is *LWP* + *ResNet*34 without an attention mechanism. Since the dual-attention (DA) module contains two sub-attention modules: self-attention (SA) and channel-attention (CA), we conducted four models: BS, BS + SA, BS + CA, and BS + DA.

Table 6 shows the performance of the attention mechanism ablation study. The results show that the dual-attention module performs better than others.

#### 3.2.5. Visualization

**Figure 7.**
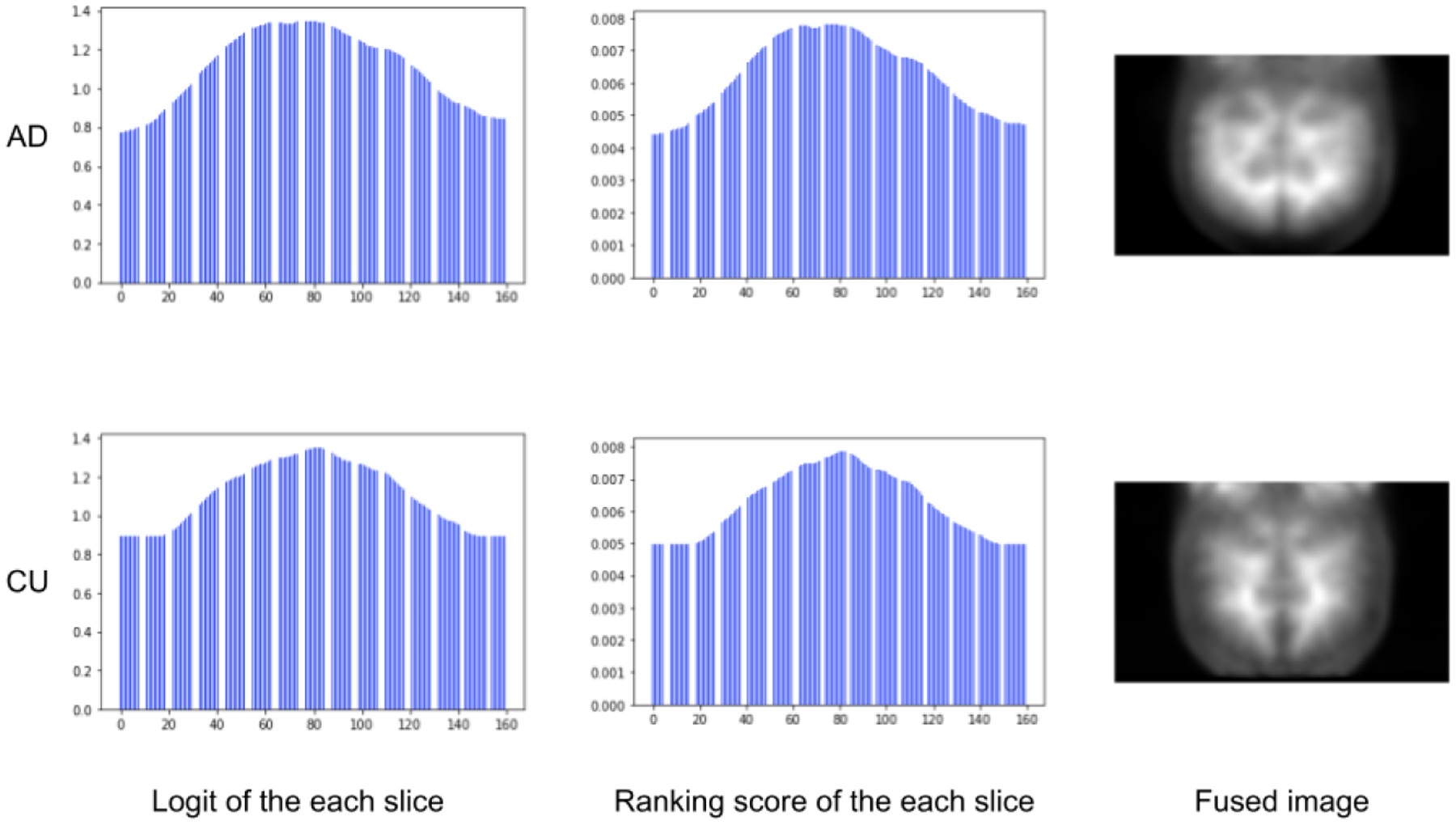
The visualization results of the PET image. The first column shows the digit of each slice. The second column shows the rank score of the slices after the softmax function. The last column shows the fused PET image.

Figure 7 shows the results on the slice logits, slice ranking scores, and fused images. We found that central slices of the 3D brain outweighed the surrounding slices. It indicates that brain regions covered by the central slices may play a more important role in revealing AD pathology than those in the lateral slices.

## 4. Discussion

In this study, we have proposed a novel method for training the 3D image by 2D CNN and end-to-end network architecture. We showed that our newly developed model significantly reduced the processing time while achieving comparable performance compared with traditional 3D CNN models. We also demonstrated several other novel findings, as follows. First, we found that ResNet34 outperforms other 2D CNN backbones as a feature extractor. Second, we demonstrated that different views of 2D images may have different performing outcomes when converting 3D images into 2D. In the current study, we showed that the coronal view performed better than the other two. Third, we showed that the LWP model can effectively convert the 3D to 2D fused images with low training time and computation cost while maintaining high performance. The visualized results further illustrated that the mid-range slices had higher importance than the side-range slices. Fourth, we have proposed a new attention module by paralleling the self-attention module and channel-wise attention module together for better discriminative feature extraction. Our results demonstrated the effectiveness of the new attention module.

Our method is inspired by the ARP method but it is different in implementation. The ARP method fuses the slices of the 3D image with the static weights, but our method is data-driven and learnable by the model itself. Compared with ARP, the LWP can capture more informative features and can gain and boost better performance. Compared with the traditional 3D CNN models, the LWP method saves much more training time and computation cost and achieves comparable or even better performance. Compared with conventional 2D CNN models, the fused image of LWP contains more informative features than the single-slice 2D images, which show better performance than the single-slice input of the 2D CNN. The idea of the LWP method is to record the abnormal variance between the 3D brain slices. In the medical domain, there is severe shrinkage in the structure and different metabolism densities of the brain in an AD patient. The 3D-to-2D projection concentrates on extracting the discriminative image-level information between CU and AD is the critical input of the 2D CNN.

The newly developed 3D-2D model may have profound implications in the future clinical setting for AD early diagnosis. Currently, 3D brain images are not widely available or used in routine clinical diagnosis. One of the major reasons is the unreasonably long processing time needed to get the information timely in daily health care. The proposed model needs fewer computation resources than the traditional 3D models, making the computation faster and less required for the hardware demands, which may be more applicable and affordable to be implemented in the clinical setting, as well as for mobile device use, in the future. Our future work will apply this method to other 3D brain image modalities, such as MRI structural and cerebral blood flow imaging analysis.

A limitation of the study is the lack of the spatial structure information of the 3D image as there was a trade-off between the 3D image spatial information and computation efficiency in our model. Further, our focus in the present study was on the classification of late stage AD versus CU and using PET-AV45 imaging only. In the future, it will be important to apply a similar method to earlier clinical stages (e.g., early mild cognitive impairment) and include other imaging measurements, such as brain atrophy using MRI.

In conclusion, we demonstrated a novel 3D-2D CNN conversion model which significantly increased the efficiency of Alzheimer’s disease classification using PET-AV45 imaging. The method may have important implications for disease diagnosis and medical applications using mobile devices in the future.

## Figures and Tables

**Table 1.** Demographic of the study data.

	Num	Age	Gender (F)	Edu. (Y)	Race (W)	APOE *ϵ*4
CU	214	73.6 ± 6.0	52.2%	16.5 ± 2.6	90.2%	26.6%
AD	167	74.7 ± 8.4	41.8%	15.7 ± 2.6	90.4%	65.8%
*p*-value		0.1511	0.0974	0.0017*	0.7346	<0.0001 *

CU, cognitively unimpaired; AD, Alzheimer’s disease. Values are displayed as the mean ± SD.

Asterisk (*) next to *p*-value indicates statistical significance.

**Table 2.** The performance results of different backbone models on PET image.

Model	Acc	AUC	F1	Precision	Recall	AP
MobileNet_v2	0.8346	0.8745	0.8085	0.8210	0.7964	0.7431
VggNet11	0.8478	0.8851	0.8187	0.8562	0.7844	0.7661
DenseNet121	0.8477	0.8807	0.8284	0.8187	0.8383	0.7572
EfficientNet	0.8479	0.8913	0.8263	0.8263	0.8263	0.7590
ResNet34	0.8688	0.9058	0.8503	0.8503	0.8503	0.7886

**Table 3.** The performance results of LWP on different views of the 3D brain image.

Views	Acc	AUC	F1	Precision	Recall	AP
Axial	0.8609	0.8811	0.8328	0.8800	0.7904	0.7874
Coronal	0.8688	0.9058	0.8503	0.8503	0.8503	0.7886
Sagittal	0.8609	0.8692	0.8349	0.8701	0.8024	0.7848

**Table 4.** The performance results of different 2D and 3D CNN models on PET image.

Model	Acc	AUC	F1	Precision	Recall	AP
3D-CNN [14]	0.8482	0.8797	0.8095	0.8151	0.8041	0.7339
2D-CNN [17]	0.8241	0.8446	0.8069	0.7778	0.8383	0.7229
Jo et al. [15]	0.8451	0.8649	0.8162	0.8506	0.7844	0.7618
Hybrid [19]	0.8714	0.8654	0.8537	0.8512	0.8563	0.7919
Max.	0.8583	0.8787	0.8333	0.8599	0.8084	0.7791
Avg.	0.8661	0.8904	0.8440	0.8625	0.8263	0.7888
ARP [24]	0.8609	0.8811	0.8349	0.8701	0.8024	0.7848
LWP(current study)	0.8871	0.9088	0.8693	0.8827	0.8563	0.8189

**Table 5.** The training computation cost of different 2D and 3D CNN models on PET image.

Model	Batch	MADs (G)	e-Time(s)
3D-CNN [14]	8	96.02	56.27 ± 1.45*
2D-CNN [17]	16	0.19 × 10^−3^	2.03 ± 0.03
Jo et al. [15]	16	34.39	36.18 ± 0.05
Hybrid [19]	16	1.86	2.16 ± 0.04
Max.	16	18.15	14.33 ± 0.12
Avg.	16	18.15	14.02 ± 0.63
ARP [24]	16	18.15	17.14 ± 0.02
LWP (current study)	16	18.53	16.69 ± 0.05

Asterisk (*) indicates the largest value of the epoch time (e-Time).

**Table 6.** The performance results of ablation study on attention mechanism.

Models	Acc	AUC	F1	Precision	Recall	AP
BS	0.8688	0.9058	0.8503	0.8503	0.8503	0.7886
BS + SA	0.8766	0.9135	0.8563	0.8750	0.8383	0.8044
BS + CA	0.8740	0.9040	0.8537	0.8696	0.8383	0.7998
BS + DA	0.8871	0.9178	0.8693	0.8827	0.8563	0.8189

## Data Availability

Data used in this article were collected from the Alzheimer’s Disease Neuroimaging Initiative (ADNI) database (adni.loni.usc.edu). The collected data are available here: https://drive.google.com/file/d/1LntBRn_STYdBOJAN2V1RWws1Ih1ACsmC/view?usp=sharing (accessed on 9 January 2023).
